# Comparison of clinico-epidemiological and radiological features in paracoccidioidomycosis patients regarding serological classification using antigens from *Paracoccidioides brasiliensis* complex and *Paracoccidioides lutzii*

**DOI:** 10.1371/journal.pntd.0008485

**Published:** 2020-08-25

**Authors:** Edy F. Pereira, Gregory Gegembauer, Marilene R. Chang, Zoilo P. de Camargo, Thiago F. Nunes, Sergio M. Ribeiro, Lídia R. de Carvalho, Bianca M. Maldonado, Rinaldo P. Mendes, Anamaria M. M. Paniago

**Affiliations:** 1 Federal University of Mato Grosso do Sul (UFMS), Campo Grande, Mato Grosso do Sul state, Brazil; 2 Department of Microbiology, Immunology and Parasitology, Cell Biology Division, Federal University of São Paulo (UNIFESP), São Paulo, São Paulo state, Brazil; 3 Faculdade de Medicina de Botucatu, São Paulo State University (UNESP), Botucatu, São Paulo state, Brazil; 4 Institute of Biosciences, Botucatu—São Paulo State University (UNESP), Botucatu, São Paulo state, Brazil; Universidad de Antioquia, COLOMBIA

## Abstract

Genotyping of the genus *Paracoccidioides* showed its diversity and geographical distribution. Four species constituting the *Paracoccidioides brasiliensis* complex and *Paracoccidioides lutzii* are etiological agents of paracoccidioidomycosis (PCM). However, there are no studies comparing the clinical and epidemiological aspects between PCM caused by the *P*. *brasiliensis* complex and by *P*. *lutzii*. Demographic and clinical data from 81 patients with PCM—confirmed by mycological and/or histopathological examination—from Mato Grosso do Sul state (Brazil) were studied. All patients underwent serology by immunodiffusion with antigens obtained from the *P*. *brasiliensis* complex (Exo*Pb* and gp43) and Cell Free Antigens obtained from *P*.*lutzii* (CFA*Pl*).The cases were classified regarding their serological profile into three groups: G1: PCM patients seropositive to Exo*Pb* and/or gp43 and seronegative to CFA*Pl* (n = 51), assumed to have PCM caused by *P*. *brasiliensis* complex; G2: PCM patients seronegative to gp43 and seropositive to CFA*Pl* (n = 16), with PCM caused by *P*. *lutzii*; and G3: PCM patients seropositive to Exo*Pb* or gp43 and seropositive to CFA*Pl* (n = 14), with undetermined serological profile, was excluded from the analyses. The Fisher's exact test or the Mann-Whitney U test, and cluster analysis according to Ward’s method and Euclidean distance were used to analyze the results. Patients with serological profile suggestive of *P*. *lutzii* lived predominantly in municipalities in the Central and Southern regions of the state, while those with serological profile indicative of the *P*. *brasiliensis* complex were distributed throughout the state. No differences were found between the two groups regarding gender, age, schooling, rural work, clinical form, severity, organs involved, intensity of pulmonary involvement, degree of anemia, erythrocyte sedimentation rate values, and therapeutic response. PCM patients with serological profile suggestive of *P*. *lutzii* and PCM patients with serological profile indicative of *P*. *brasiliensis* complex showed the same clinical and radiological presentations.

## Author summary

Paracoccidioidomycosis (PCM) is a disease caused by fungi living in the soil, which are inhaled mainly by rural workers from Latin America. These fungi are classified into the *Paracoccidioides brasiliensis* complex and *Paracoccidioides lutzii*. PCM may involve any organ and its treatment frequently results in fibrotic scars and functional limitations, which not rarely lead to early retirement. The mycological examination performed by routine laboratory tests cannot identify these species. However, the serological tests can suggest what species the etiological agent is, long as an antigen from both species is used. In order to determine whether there are differences in epidemiological aspects, clinical manifestations, severity, treatment response, and intensity of lung lesions according to the species involved, we compared 51 patients with serological profile suggestive of PCM caused by *P*. *brasiliensis* complex with 16 others caused by *P*. *lutzii*. Our results showed no differences between these species related to the parameters evaluated. Nevertheless, other clinical variables should be evaluated to detect differences regarding species. This study reinforces the importance of using antigens from both species for serological tests in PCM diagnosis, determination of severity, treatment follow-up and control of cure.

## Introduction

Paracoccidioidomycosis (PCM) is a systemic disease caused by thermo-dimorphic fungi *Paracoccidioides*, and it is the most significant endemic mycosis of Latin America- especially in Brazil, Venezuela, Colombia, and Argentina- because of its high incidence [1]. PCM patients present an antigen-specific immunosuppression, which can be recovered after appropriate treatment [2, 3]

Several species of the genus *Paracoccidioides* involved in PCM etiology have been identified, such as *P*. *brasiliensis*, *P*. *americana*, *P*. *restrepiensis*, and *P*. *venezuelensis*. Collectively, these constitute the *P*. *brasiliensis* species complex. *P*. *lutzii*, which does not belong to this species complex, has also been identified as an etiological agent of PCM [4, 5].

Accurate identification of these agents depends on molecular evaluation of clinical specimens. However, this is infeasible because of the slow fungal growth, low frequency of *Paracoccidioides* spp isolation in culture, and because the molecular identification of fungi is not performed to routine laboratories. Therefore, it has been proposed to use specific *P*. *lutzii* antigens to perform serological tests in PCM so as to differentiate between its two main causative species [6].

Although only a few studies have been conducted to determine the exact geographical distribution of *P*. *lutzii* in Brazil, it is known to predominate in the Midwest Region of Brazil, [7, 8]. PCM caused by *P*. *lutzii* is known to occur in Mato Grosso do Sul [6]. This state is in the Midwest region of Brazil and is the neighboring state of Mato Grosso, where many *P*. *lutzii* cases have been reported previously [9], despite the absence of information on the geographical distribution in the State.

Since the identification of *P*. *lutzii*, it has been questioned whether it causes a different clinical presentation of PCM compared to *P*. *brasiliensis* infection.

This study aimed to compare the demographical, clinical and radiological aspects of PCM cases serologically identified as caused by the *P*. *brasiliensis* complex with cases caused by *P*. *lutzii* in Mato Grosso do Sul state.

## Methods

### Study design, setting and patients

The present study was conducted from March 2008 to March 2012 with PCM patients monitored by the Reference Service for Infectious and Parasitic Diseases at the University Hospital of Federal University of Mato Grosso do Sul, Campo Grande, Mato Grosso do Sul state, Brazil.

The inclusion criteria for this study were as follows: a confirmed laboratory diagnosis of PCM characterized by the identification of the typical *Paracoccidioides* spp. yeast forms in clinical samples by mycological or histopathological examinations from patients presenting symptoms consistent with active infection. Clinical, sociodemographic, and laboratory variables were prospectively recorded on a standardized form. Such variables were compared between two different group of patients, i. e., a group with PCM presumably caused by the *P*. *brasiliensis* complex and another group with PCM supposedly caused by *P*. *lutzii*.

Thirty-four patients in this study participated in a previous study about serology of PCM due to *P*. *lutzii* [6] and 60 patients participated in a study about prevalence of the acute/subacute PCM in Mato Grosso do Sul [10].

### Definition of study groups

Serology was performed in all patients included at the Immunology Laboratory of the Federal University of São Paulo (UNESP), São Paulo state, Brazil. Three antigenic preparations—(1) *P*. *brasiliensis* exoantigen (Exo*Pb*); (2) purified 43 kDa glycoprotein (gp43); (3) Cell Free Antigen from *P*. *lutzii* isolate (CFA*Pl*)—were used for the detection of specific antibodies via double immunodiffusion (DID) in agar gel.

The Exo*Pb* and gp43 antigens obtained from B339 isolates and the CFA*PI* antigen obtained from EPM 208 isolates were identified through polymerase chain reaction (PCR) as *P*. *brasiliensis* and *P*. *lutzii*, respectively. The *P*. *brasiliensis* complex was differentiated from *P lutzii* using the primers HSPMMT1 and PLMMT1 as molecular markers [4].

DID was conducted according to the modified Ouchterlony´s method [11]. For every sample, tests were performed with each one of the antigens: Exo*Pb*, gp43, and CFA*Pl*.

Depending on the antigen identification by serum antibodies, patients were distributed into 3 groups: group1: PCM patients seropositive to Exo*Pb* and/or gp43 and seronegative to CFA*Pl*, with 51 patients; group 2: PCM patients seronegative to gp43 and seropositive to CFA*Pl*, with 16 patients; and group 3: PCM patients seropositive to Exo*Pb* or gp43 and seropositive to CFA*Pl*, with 14 patients.

Group 1 patients were serologically compatible with the *P*. *brasiliensis* complex and group 2 with *P*. *lutzii* etiology. Finally, PCM patients in group 3 have not a determined etiology and they were excluded from the comparative analysis.

**Fig 1:**
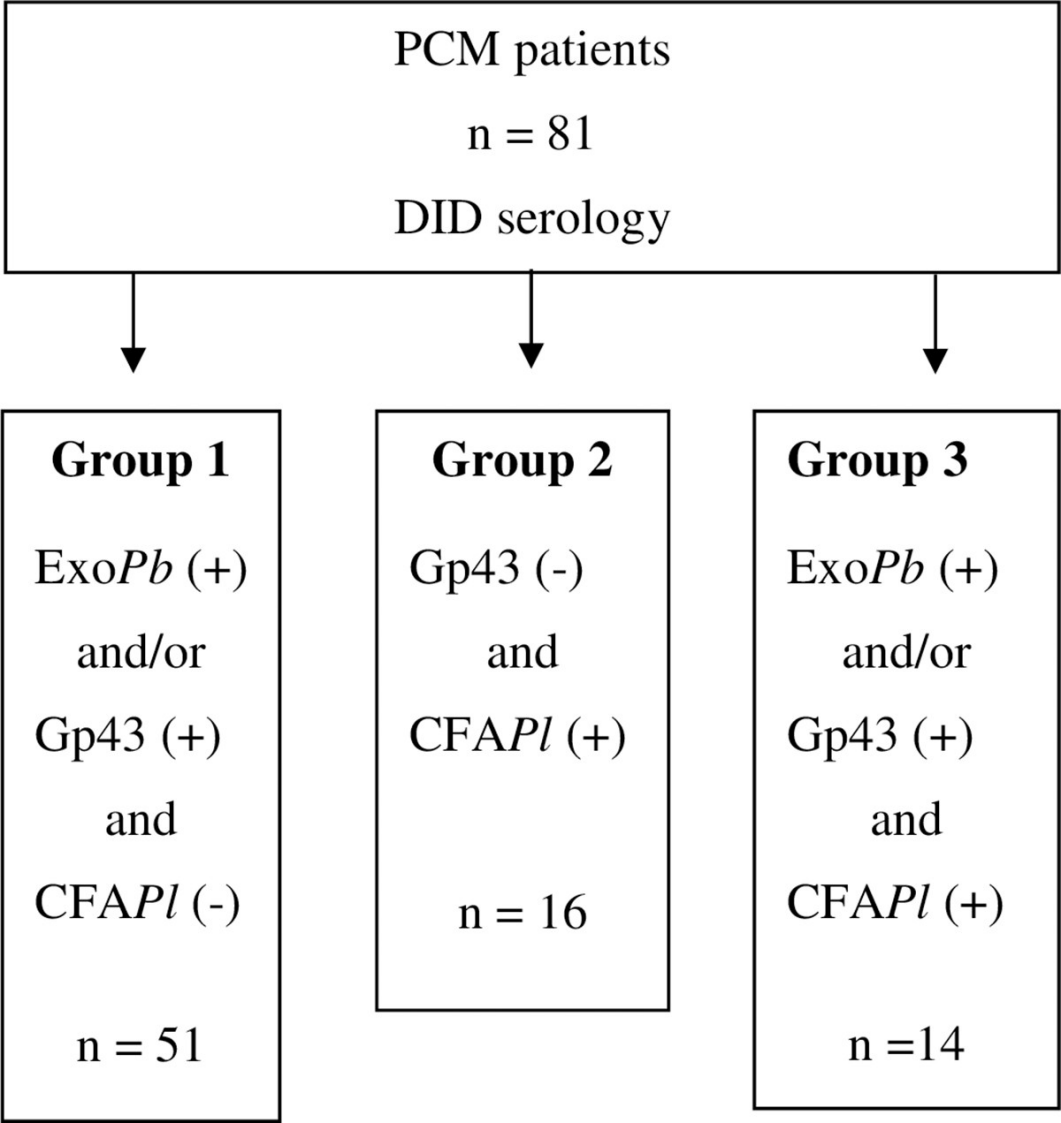
Flow diagram of PCM cases and their serological results. DID: Double agar gel immunodiffusion test; Exo*Pb*: *P*. *brasiliensis* exoantigen; gp43: purified 43 kDa glycoprotein; CFA*Pl*: Cell free antigen from *P*. *lutzii*.

The flow diagram (Fig 1) summarizes the number of subjects enrolled and included in the study.

### Sample size

Sample size calculation was performed for difference between proportions, considering an error type α of 0.05, 80% test power, and 35% differences, according to Jekel et al. (2005) [12]. Every arm of this study should have at least 16 patients.

### Prevalence rate

Prevalence rates were calculated by dividing the number of cases treated at the study site, which is a reference for PCM, by the number of inhabitants in the state in 2010. We employed such year as the cases were diagnosed between 2008 and 2012, and the number of inhabitants was estimated on the records of the Brazilian Institute of Geographic and Statistic (IBGE), 2010 survey [13]

### Demographic and clinical data

Demographic and clinical data were collected prospectively using a standardized protocol.

PCM patients were classified into acute/subacute or chronic forms. The acute/subacute form was subdivided into moderate and severe forms, and the chronic form into mild, moderate, and severe forms [1, 14]. The criteria used in the classification of severity were weight loss, dissemination of the disease, intensity of pulmonary involvement, serum levels of specific antibodies, and degree of lymph node enlargement [1, 14].

Antifungal drugs were administered according to the Brazilian guidelines [15]. The follow-up regimen included reevaluation after 1 month of treatment, and every 2 months thereafter until clinical cure was reached. Clinical cure was defined as the disappearance of the previously detected symptomatology and regression of the erythrocyte sedimentation rate or C-reactive protein to normal values [1]. Patients were then evaluated every 2–3 months until serological cure was reached.

**Fig 2:**
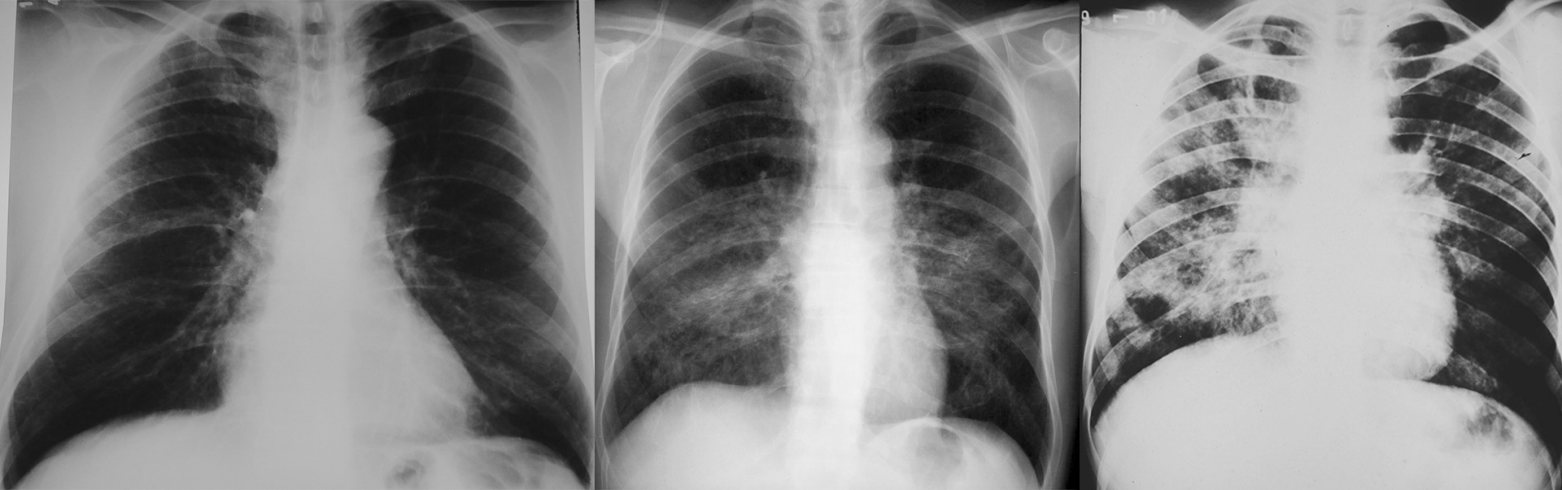
Chest roentgenograms of three cases. From left to right: mild, moderate, and severe.

The severity of pulmonary involvement was assessed based on standard posteroanterior and lateral views of chest roentgenograms obtained at patient admission. The same radiologist analyzed all the radiographs and classified the severity of pulmonary involvement into mild, moderate or severe: a) mild–only one or two focal pulmonary lesions, or alveolar lesions even if involving more than one third of the total pulmonary parenchyma; b) moderate–three or more focal lesions were present, or interstitial or mixed lesions involving less than one third of the total lung parenchyma; c) severe–interstitial or mixed lesions involving more than one third of the total lung parenchyma. Fig 2 illustrates the radiography of mild, moderate and severe cases (Fig 2) [16].

### Statistical analysis

The Fisher's exact test was used to compare categorical variables, and the Mann-Whitney *U* test to compare continuous variables. To study the similarities in prevalence rate between different municipalities, cluster analysis was performed according to Ward’s method and Euclidean distance. Significance was set up at p≤0.05. The software used was SPSS version 21.0 (IBM Corporation, Chicago, IL, USA).

### Ethical considerations

The Ethics Committee of the Federal University of Mato Grosso do Sul approved the present study with ID number 354898. All patients signed a statement of written informed consent for data collection. In the cases of minors, their parents or legal guardians did it on their behalf.

## Results

All 81 patients included have confirmed PCM. In 57 (70.4%) of them, the confirmation was by histopathological examination; in 13 (16.0%) of them, it was by direct mycological examination; and in the other 11 patients (13.6%), it was by both examinations.

Out of the 81 PCM patients studied, 51 (62.9%) had PCM serologically compatible with the *P*. *brasiliensis* complex etiology (group 1) and16 (19.8%) had it compatible with *P*. *lutzii* etiology (group 2). In the remaining 14 (17.3%), the species was serologically undetermined (group 3).

Except for ethnicity, no significant differences in the demographic variables were found between group1 and group 2, as shown in Table 1.

**Table 1:** Demographic findings of 81 paracoccidioidomycosis patients according to their serological profile.

Variable	Group 1 n = 51 Nº (%) or median [range]	Group 2 n = 16 Nº (%) or median [range]	Group 3 n = 14 Nº (%) or median [range]	Group 1 vs Group2 *p*
**Gender**				
Male	48 (94.1)	15 (93.8)	14 (100)	1.00^#^
Female	3 (5.9)	1 (6.2)	0	
**Ethnicity**				0.05^#^
White	28 (54.9)	3 (18.8)	4 (28.6)	0.03^&^
Black	3 (5.9)	1 (6.2)	0	0.57^&^
Mixed	16 (31.4)	10 (62.5)	7 (50,0)	0.83^&^
Indigenous	0	0	1 (7.1)	
Unknown*	4 (7.8)	2 (12.5)	2 (14.3)	
**Age** (years)	47.4 [4–76]	49.5 [35–66]	51 [32–69]	0.89^†^
**Education** (years)				0.71^#^
≤ 4	29 (56.9)	9 (56.2)	13 (92.9)	
> 4	8 (15.7)	4(25.0)	1 (7.1)	
Unknown*	14 (27.4)	3 (18.8)	0	
**Rural workers**				0.67^#^
Yes	41 (80.4)	15 (93.8)	14 (100)	
No	6 (11.8)	1 (6.2)	0	
Unknown*	4 (7.8)	0 (0.0)	0	

Group1: PCM patients seropositive to Exo*Pb* and/or gp43 and seronegative to CFA*Pl*. Group 2: PCM patients seronegative to gp43 and seropositive to CFA*Pl*. Group 3: PCM patients seropositive to Exo*Pb* or gp43 and seropositive to CFA*Pl*.

* It was not included in the statistical analysis.

#: Fisher´s Exact test.

&: comparison of two proportions test.

†: Mann-Whitney *U* test.

**Fig 3:**
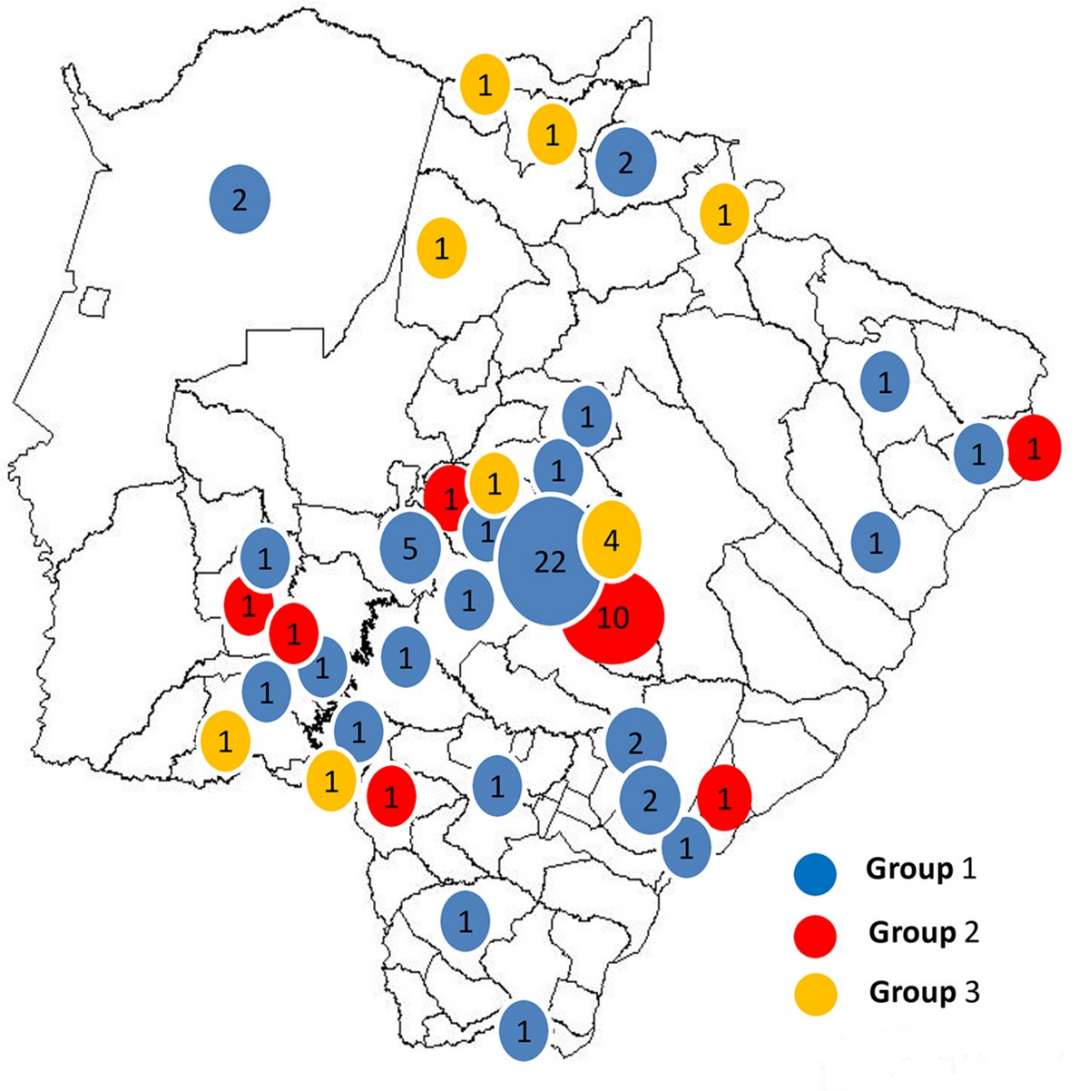
Distribution of 78 cases of paracoccidioidomycosis from the Mato Grosso do Sul state.

**Fig 4:**
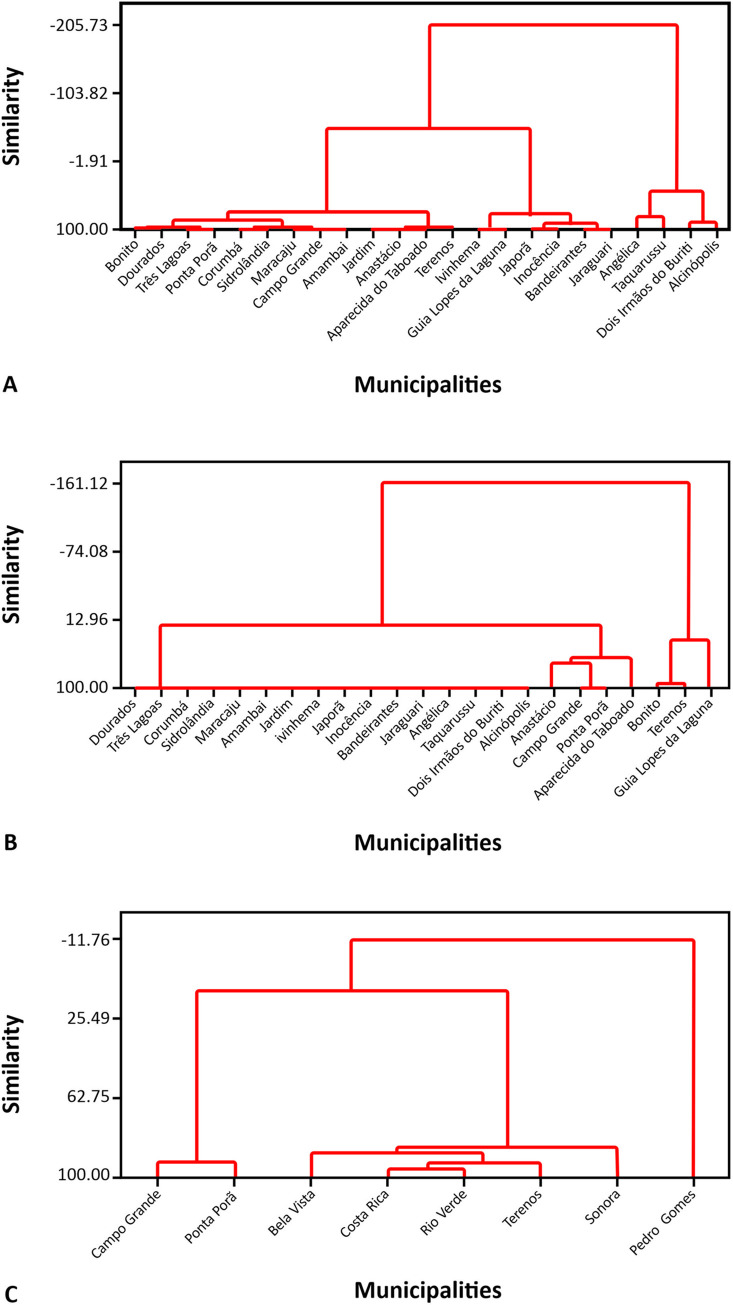
Dendrograms related to the prevalence rates of paracoccidioidomycosis (PCM) per 100,000 inhabitants, January 2008—December 2012. A. Group 1, PCM patients seropositive to gp43 and/or Exo*Pb* and seronegative to CFA*Pl* (51 cases). B. Group 2, PCM patients seronegative to gp43 and Exo*Pb* and seropositive to CFA*Pl* (16 cases). C. Group 3, PCM patients seropositive to gp43 or Exo*Pb* and seropositive to CFA*Pl* (11 cases). The reference year for the population was 2010.

The distribution of cases according to the municipality of origin is shown in Fig 3 (Fig 3). It is observed that most patients live in the center of the State. This distribution, according to the prevalence rate and similarities, is presented in Table 2 and Fig 4. Three cases of the group 3 were not from Mato Grosso do Sul, being one from São Paulo state, another from Mato Grosso and the other one from Paraguay.

**Table 2:** PCM cases and prevalence rate (PR) per 100,000 inhabitants according to their municipalities and serological profiles.

State/Municipalities	Population (year 2010)	Group 1 n (PR)	Group 2 n (PR)	Group 3 n (PR)
**Mato Grosso do Sul**	**2,449,024**	**51 (2.1)**	**16 (0.7)**	**11 (0.4)**
**Campo Grande**	786,797	22 (2.8)	10 (1.3)	4 (0.5)
**Dois Irmãos do Buriti**	10,363	5 (48.2)	0 (0.0)	0 (0.0)
**Alcinópolis**	4,569	2 (43.8)	0 (0.0)	0 (0.0)
**Angélica**	9,185	2 (21.8)	0 (0.0)	0 (0.0)
**Corumbá**	103,703	2 (1.9)	0 (0.0)	0 (0.0)
**Ivinhema**	22,341	2 (9.0)	0 (0.0)	0 (0.0)
**Amambaí**	34,730	1 (2.9)	0 (0.0)	0 (0.0)
**Anastácio**	23,835	1 (4.2)	0 (0.0)	0 (0.0)
**Aparecida do Taboado**	22,320	1 (4.5)	1 (4.5)	0 (0.0)
**Bandeirantes**	6,609	1 (15.1)	0 (0.0)	0 (0.0)
**Dourados**	196,035	1 (0.5)	0 (0.0)	0 (0.0)
**Guia Lopes da Laguna**	10,366	1 (9.6)	1 (9.6)	0 (0.0)
**Inocência**	7,669	1 (13.0)	0 (0.0)	0 (0.0)
**Japorã**	7,731	1 (12.9)	0 (0.0)	0 (0.0)
**Jaraguari**	6,341	1 (15.8)	0 (0.0)	0 (0.0)
**Jardim**	24,346	1 (4.1)	0 (0.0)	0 (0.0)
**Maracaju**	37,405	1 (2.7)	0 (0.0)	0 (0.0)
**Ponta Porã**	77,872	1 (1.3)	1 (1.3)	1 (1.3)
**Sidrolândia**	42,132	1 (2.4)	0 (0.0)	0 (0.0)
**Taquarussu**	3,518	1 (28.4)	0 (0.0)	0 (0.0)
**Terenos**	17,146	1 (5.8)	1 (5.8)	1 (5.8)
**Três Lagoas**	101,791	1 (1.0)	0 (0.0)	0 (0.0)
**Batayporã**	10,936	0 (0.0)	1 (9.1)	0 (0.0)
**Bonito**	19,587	0 (0.0)	1 (5.1)	0 (0.0)
**Bela Vista**	23,181	0 (0.0)	0 (0.0)	1 (4.3)
**Costa Rica**	19,695	0 (0.0)	0 (0.0)	1 (5.1)
**Pedro Gomes**	7,967	0 (0.0)	0 (0.0)	1 (12.6)
**Rio Verde de Mato Grosso**	18,890	0 (0.0)	0 (0.0)	1 (5.3)
**Sonora**	14,833	0 (0.0)	0 (0.0)	1 (6.7)

Group1: PCM patients seropositive to Exo*Pb* and/or gp43 and seronegative to CFA*Pl*. Group 2: PCM patients seronegative to gp43 and seropositive to CFA*Pl*. Group 3: PCM patients seropositive to Exo*Pb* or gp43 and seropositive to CFA*Pl*.

The highest rates of the disease serologically compatible with the *P*. *brasiliensis* complex etiology were found in Dois Irmãos do Buriti (48.2/100,000 inhabitants) and Alcinópolis (43.8/100,000 inhabitants), while the highest rates involving *P*. *lutzii* were observed in Guia Lopes da Laguna (9.6/100,000 inhabitants) and Batayporã (9.1/100,000 inhabitants).

An evaluation of group 1 PCM patients serologically compatible with the *P*. *brasiliensis* complex etiology reveals the formation of three geographic groups of municipalities, according to the similarity of prevalence: the first one, made up of Bonito, Dourados, Três Lagoas, Ponta Porã, Corumbá, Sidrolândia, Maracaju, Campo Grande, Amambai, Jardim, Anastácio, Aparecida do Taboado, and Terenos; the second one, constituted by Ivinhema, Guia Lopes da Laguna, Japorã, Inocência, Bandeirantes and Jaraguari; and the third one, composed of Angélica, Taquarussu, Dois Irmão do Buriti and Alcinópolis (Fig 4A).

Fig 4B (Fig 4B) reveals the formation of two groups of municipalities according to the prevalence rates of PCM patients, serologically compatible with *P*. *lutzii* etiology: the first one, constituted by Dourados, Três Lagoas, Corumbá, Sidrolândia, Maracaju, Amambaí, Jardim, Ivinhema, Japorã, Inocência, Bandeirantes, Jaraguari, Angélica, Taquarussu, Dois Irmãos do Buriti, Alcinópolis, Anastácio, Campo Grande, Ponta Porã, and Aparecida do Taboado; and the second, formed by Bonito, Terenos, and Guia Lopes da Laguna.

In sequence, an evaluation of group 3 with PCM patients by serologically undetermined species, reveals the formation of two geographic groups according of prevalence rate: the first one, made up by Campo Grande, Ponta Porã, Bela Vista, Costa Rica, Rio Verde, Terenos and Sonora; and the second one, constituted by Pedro Gomes (Fig 4C).

Group1 and 2 did not differ regarding clinical form, organs involved, intensity of pulmonary involvement, treatment, and progress after treatment (Table 3).

**Table 3:** Clinical aspects of 81 paracoccidioidomycosis patients according to their serological profiles.

Variables	Group 1N° (%) or median [range]	Group 2N° (%) or median [range]	Group 3 N° (%) or median [range]	Group 1 vs Group2 *p*
**Clinical form/severity (n = 81)**				1.00
Acute/subacute	5 (9.8)	1 (6.3)	- (-)	1.00
Moderate	2 (40.0)	1 (100.0)	(-)	
Severe	3 (60.0)	- (-)		
Chronic	46 (90.2)	15 (93.8)	14 (100.0)	0.41
Mild	13 (28.3)	2 (13.3)	1	
Moderate	22 (47.8)	10 (66.7)	8	
Severe	10 (21.7))	3 (20.0)	5	
Unclassified*	1 (2.2)	- (-)	- (-)	
**Lesion site (n = 81)**				
Lungs	45 (88.2)	14 (87.5)	14 (100.0)	1.0
Oral cavity and/or oropharynx	26 (51.0)	12 (75.0)	9	0.15
Larynx	11 (21.6)	2 (12.5)	5	0.72
Lymph node	11 (21.6)	6 (37.5)	7	0.21
Central nervous system	4 (7.8)	1 (6.3)	2	1.00
Adrenal glands	7 (13.7)	3 (18.8)	1	0.69
**Intensity of lung lesions, on chest X-ray- (n = 41)**			NA	0.90
Mild	16 (53.3)	5 (45.4)		
Moderate	9 (30.0)	4 (36.4)		
Severe	5 (16.7)	2 (18.2)		
**Albumin (g/dL) (n = 50)**	3.8 [2.0–5.1]	3.3 [1.3–3.9]	3.5 [1.4–4.5]	0.14
**Hemoglobin (g/dL) (n = 72)**	13.8 [8.5–16.6]	13.1 [9.5–19.0]	13.9 [9.7–16.4]	0.67
**ESR (mm/hour) (n = 56)**	23 [1.0–117.0]	61.5 [2.0–90.0]	50.0 [3.0–115.0]	0.17
**Antifungal compound (n = 77)**				
Cotrimoxazole	40 (80.0)	15 (93.7)	12 (85.7)	0.43
Itraconazole	8 (16.0)	1 (6.3)	2 (14.3)	
Amphotericin B*	1 (2.0)	- (-)	-	
Fluconazole*	1 (2.0)	- (-)	-	
**Completed treatment (n = 80)**				0.26
Yes	22 (44.0)	10 (62.5)	8 (57.1)	
No	28 (56.0)	6 (37.5)	6 (42.9)	
**Death (n = 81)**				1.00
Yes	1 (2.0)	- (-)	- (-)	
No	50 (98.0)	16 (100)	14 (100.0)	
**Relapse (n = 41)**				0.65
Yes	4 (18.2)	3 (30.0)	1 (7.1)	
No	18 (81.8)	7 (70.0)	13 (82.9)	
**Cure with pulmonary sequelae (n = 41)**				0.61
Yes	19 (86.4)	8 (80.0)	7 (87.5)	
No	3 (13.6)	2 (20.0)	1 (7.1)	

* It was not included in the statistical analysis

Group1: PCM patients seropositive to Exo*Pb* and/or gp43 and seronegative to CFA*Pl*. Group 2: PCM patients seronegative to gp43 and seropositive to CFA*Pl*. Group 3: PCM patients seropositive to Exo*Pb* or gp43 and seropositive to CFA*Pl*. * It was not included in the statistical analysis: Fisher´s Exact test to compare categorical variables. Mann-Whitney *U* test to compare numerical variables. NA: not available

## Discussion

The recent identification of two different PCM-causing agents, the *P*. *brasiliensis* complex and *P*. *lutzii* [4, 5] raises the issue of whether there are clinical and epidemiological differences in the disease caused by them. While this study was ongoing, additional PCM-causing species were identified: *P*. *americana*, *P*. *restrepiensis*, *and P*. *venezuelensis*. Together with *P*. *brasiliensis*, these fungal species now form the *Paracoccidioides brasiliensis* complex [5].

The reference standard for *Paracoccidioides* species identification is molecular methods of the clinical isolates from culture. As *Paracoccidioides* spp growing in culture media is difficult and molecular methods are not available in routine clinical laboratory, we used serology as the strategy to presume the species causing PCM, as proposed by Gegembauer et al (2014) [6].

Our patients were classified regarding their results of immunodiffusion: group 1 with serological pattern suggestive of the *P*. *brasiliensis* complex; group 2 with serological profile suggestive of *P*. *lutzii*; and group 3 with undetermined serological profile. As the purpose of our study is to investigate differences between PCM caused by *P*. *lutzii* and PCM caused by the *P*. *brasiliensis* complex, the group 3 was excluded from the comparative analyses.

The main biochemical characteristic that differentiates *P*. *lutzii* from the *P*. *brasiliensis* complex is the absence of gp43 expression [17, 18]. Sera from patients with PCM caused by *P*. *lutzii* identified by molecular method do not react against gp43 and other antigens obtained from *P*. *brasiliensis* complex isolates, such as exo-antigen B339, which is traditionally used in the PCM immunodiagnosis. In addition, these sera have 100% reactivity against CFA obtained from *P*. *lutzii* isolates [18], and 100% sensitivity (IC 80.5–100) and 100% specificity (IC 94–100) of CFA*Pl* have been reported [6]. Although other studies show CFA*Pl* is not specific antigen for *P*. *lutzii* [17, 19], patients seropositive to CFA*Pl* and seronegative to gp43 have PCM presumably caused by *P*. *lutzii* [6]. In contrast, patients seronegative to CFA*Pl* and seropositive to gp43 have PCM presumably caused by the *P*. *brasiliensis* complex [6].

About one-fifth of the PCM cases presented a serological response profile suggestive of PCM caused by *P*. *lutzii* which occurs mainly in the Brazilian Midwest Region, having been isolated from human cases in Goiás and Mato Grosso [4, 7, 20, 21]. Mato Grosso do Sul is bordered on the north by Mato Grosso and on the northeast by Goiás, but the distribution of PCM by *P*. *lutzii* was concentrated in the south-central region of Mato Grosso do Sul. The low population density in northern Mato Grosso do Sul, where only a few cases of PCM have been reported, can justify the lack of *P*. *lutzii* cases in this area, as observed in the present study. Moreover, it should be emphasized that the place of residence of the patients may not reflect the place of infection, since most cases presented the chronic form, in which the incubation period can often extend into decades; therefore, the infection could have been acquired in another municipality or state.

Before this study, it was already speculated that at least some of the patients from Mato Grosso do Sul were infected by *P*. *lutzii*. The positivity of DID reactions performed in this study, with antigens extracted from the isolate B339 of the *P*. *brasiliensis* complex (74.5%) [22] was found to be lower than that observed in the southeast region of Brazil. This result suggested that there could be another species of the genus *Paracoccidioides* involved, similar to findings reported in a study undertaken in Mato Grosso state [21].

Two results found in the present study suggest PCM autochthony caused by *P*. *lutzii* in Mato Grosso do Sul state. First, the occurrence of an acute/subacute form in a patient serologically compatible with *P*. *lutzii* etiology, with a shorter incubation period (from a few weeks to months). Even though this patient was born in Cáceres (Mato Grosso state), where *P*. *lutzii* is quite common; it is unknown how long he lived in Campo Grande, capital of Mato Grosso do Sul state. Second, four patients with PCM serologically compatible with *P*. *lutzii* etiology were born and remained in the MS state, and reported never having left the state. Other Brazilian states have reported humans and animals infected by *P*. *lutzii*, as such as São Paulo [23] in the Southeast region; and Paraná [24] and Rio Grande do Sul [25], in South region.

The evaluation of the prevalence rates and the geographic distribution in Mato Grosso do Sul state of the three groups of patients showed no peculiarity in neither of them.

The population of Mato Grosso do Sul in 2010 was 47.6% white. In the present study, a lower percentage of white people was found within *P*. *lutzii* cases when compared to the *P*. *brasiliensis* complex cases. More than a racial susceptibility to the *Paracoccidioides* species, this finding may be related to the geographical area where the infection occurred. The agricultural areas of MS were occupied by different migratory movements. The population of the South of the country, predominantly white, has been an important colonizer in the state in recent years. Consequently, it is possible that the individual was infected with *Paracoccidioides* in his/her childhood in his/her hometown, progressing to disease after decades, due to endogenous reactivation. Out of the 20 PCM patients born in the southern region of the country, 18 (90.0%) were serologically compatible with the *P*. *brasiliensis* complex etiology.

Although the geographical distribution of *P*. *lutzii* cases is not yet defined, the methodology proposed by Gegembauer et al [6] for the classification of the species involved in PCM will certainly help to accomplish this task.

The clinical manifestations of PCM are determined by host-parasite interactions. The variable clinical presentations of PCM are usually attributed to the host's immunological status [1, 13]. Nonetheless, some intriguing regional differences related to the clinical manifestations of PCM are known, such as the absence of the acute/subacute form in the state of Rio Grande do Sul [26], in contrast to the interior São Paulo state, where cases of the acute/subacute disease forms reach 25% [27]. A number of patients with acute/subacute forms have also been reported in Goiás state [28]. Thus, it is difficult to accepted that these differences are related only to host differences and not also to environmental conditions, and consequently, to fungal species.

To the best of our knowledge, there are no publications comparing the clinical manifestations presented by PCM patients caused by agents of the *P*. *brasiliensis* complex with those from cases caused by *P*. *lutzii*. A recent publication compared findings from PCM patients caused by *P*. *brasiliensis* with those by *P*. *americana*, indicating no differences between them [29].

The present study showed no differences in the variables studied, including clinical form and organs affected, neither found any association between clinical severity and the species involved. Hahn et al [30] have recently described the clinical characteristics of 34 patients with PCM due to *P lutzii*, who presented clinical features like those previously described in cases of *P*. *brasiliensis* complex. However, no comparative evaluation was not undertaken by these authors. A report of a case of fatal fungemia caused by *P*. *lutzii* in a patient who did not exhibit any immunosuppressive co-morbidity led to the hypothesis that this species is more pathogenic than the *P*. *brasiliensis* complex [31], yet, a possible primary immunodeficiency was not investigated, as it was confirmed by Schimke et al. [32].

Therefore, although there were differences in virulence mechanism [33]; protein expression and antigenicity of *P*. *lutzii* [17, 34]; and, patterns of host-parasite interaction and pathology [35], these findings seem to have no impact on clinical manifestation, as they have on serology. Serology in PCM is particularly useful not only for diagnosis, but also severity assessment and control of cure during the treatment follow-up [1].

This study has some limitations, such as its relatively small sample size, which was enough to a safe statistical analysis, though. Another limitation is the non-molecular identification of the etiological agent. However, the serological strategy used in order to define PCM by *P*. *lutzii* could falsely identify a *P*. *brasiliensis* complex isolate as *P*. *lutzii*, since there are rare reports of molecularly confirmed PCM by *P*. *brasiliensis* complex seropositive for CFA*Pl* [19], or seronegative for gp43 [36]. and much rarer is the occurrence of both exceptions at the same time.

In conclusion, our study suggests that there are no differences in demographic aspects, pulmonary clinical and radiological manifestations, therapeutic response and progress of PCM caused by either *P*. *lutzii* or the *P*. *brasiliensis complex*. The significance of this knowledge is the maintenance of the epidemiological, clinical, radiological and therapeutic approaches, persisting the well-known differences of sensitivity of the serologic tests. Future studies, a multicentric or systematic review with meta-analysis, with a greater sample of the *Paracoccidioides* species are necessary to confirm these findings and to evaluate the importance of the routine identification of the *Paracoccidioides* species in clinical management and public health. We would like to emphasize the need to include CFA from *P*. *lutzii* in routine serological tests in endemic areas for both *Paracoccidioides* species, improving the diagnosis and allowing a better monitoring of the treatment control.

## Supporting information

S1 Checklist STROBE Checklist. (DOC)

S1 Data Dataset containing variables of the study. (XLSX)
